# Supplementary material for: Global mycorrhizal plant distribution linked to terrestrial carbon stocks
Source: Nat Commun. 2019 Nov 7;10:5077. doi: 10.1038/s41467-019-13019-2 (PMC6838125; doi:10.1038/s41467-019-13019-2)
Supplement: Supplementary file 1 — Supplementary Information [file 41467_2019_13019_MOESM1_ESM.pdf]

# Supplementary Information to the manuscript of Soudzilovskaia et al.,

# "Global mycorrhizal plant distribution linked to terrestrial carbon stocks"

Content:
Supplementary Figures 1-9
Supplementary Tables 1-5
References to the Supplementary Information

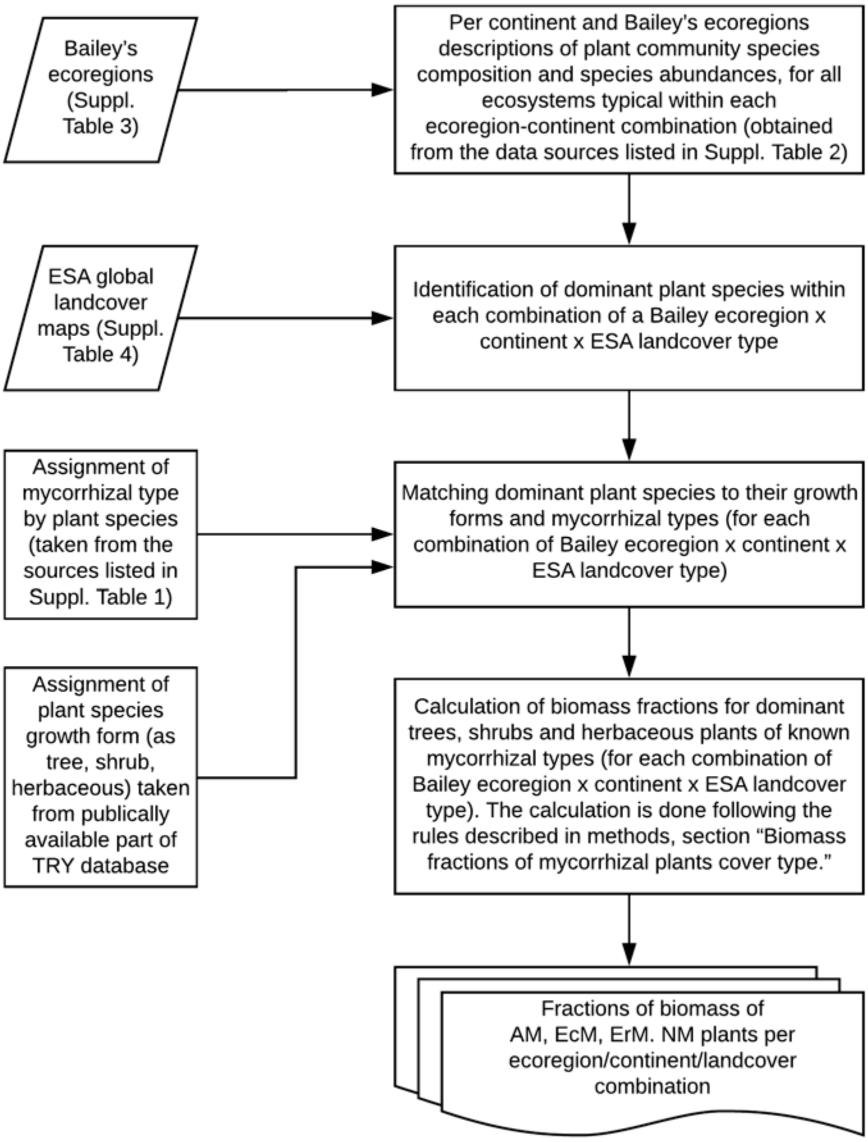


**Supplementary Figure 1. Flowchart of the data assembly processes used to construct maps of biomass fractions of plants featuring distinct mycorrhizal types.**

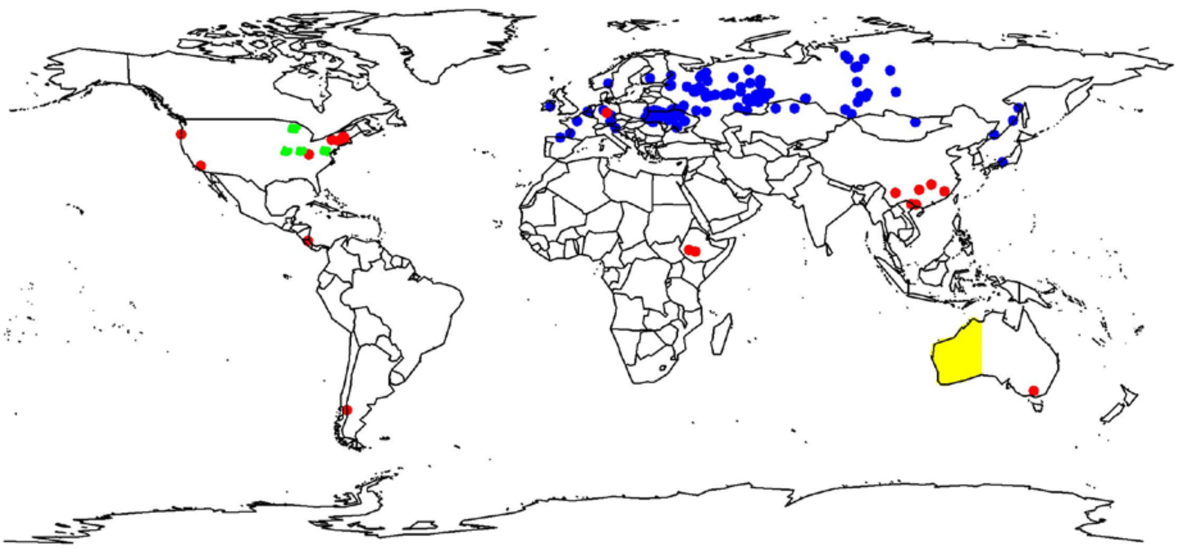

**Supplementary Figure 2. Location of the sites used for map validation. The data used are: Schepachenko and co-workers [1] (blue points), Lin et al [2] (red points), Fisher et al [3] (four areas in USA, green) and Brundrett [4] (West Australia, yellow).**

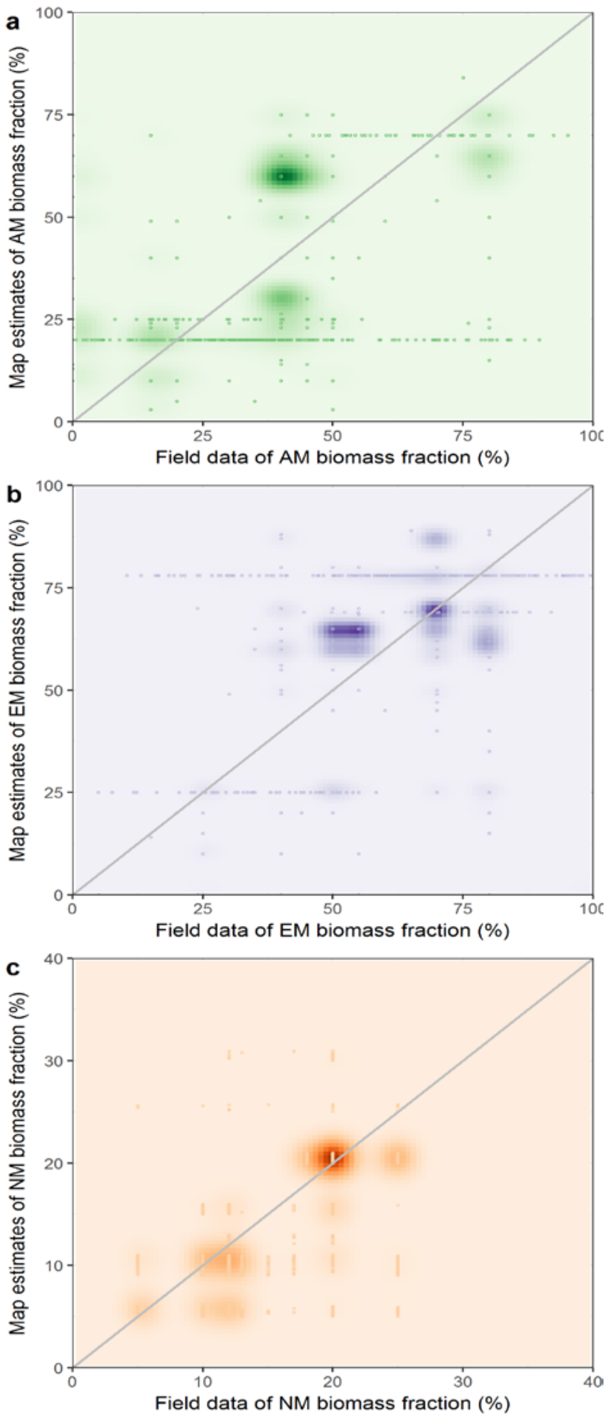


**Supplementary Figure 3. Validation of the current distribution of biomass fractions of (a) AM, (b) EcM, and (c) NM plants. The scatter plots show the relationships between the data provided in the mycorrhizal vegetation maps and the field measured data reported in the works of Schepachenko and co-workers [1] (Eurasia), Lin and co-workers [2] (global), Fisher an co-workers [3] (USA) and Brundrett [4] (West Australia). The deviation from the 1:1 line, estimated as mean averaged error (MAE), is 18.7% for AM biomass vegetation fractions, 13.6% for ECM and 4.8% for NM. $R^2$ to 1:1 line is 0.81, 0.92 and 0.93 for AM, EcM and NM biomass vegetation fractions. Due to overlap among multiple points, additionally, 2d kernel density of the probability distribution is shown with increasing colour intensity. Source data are provided as a Source Data file.**

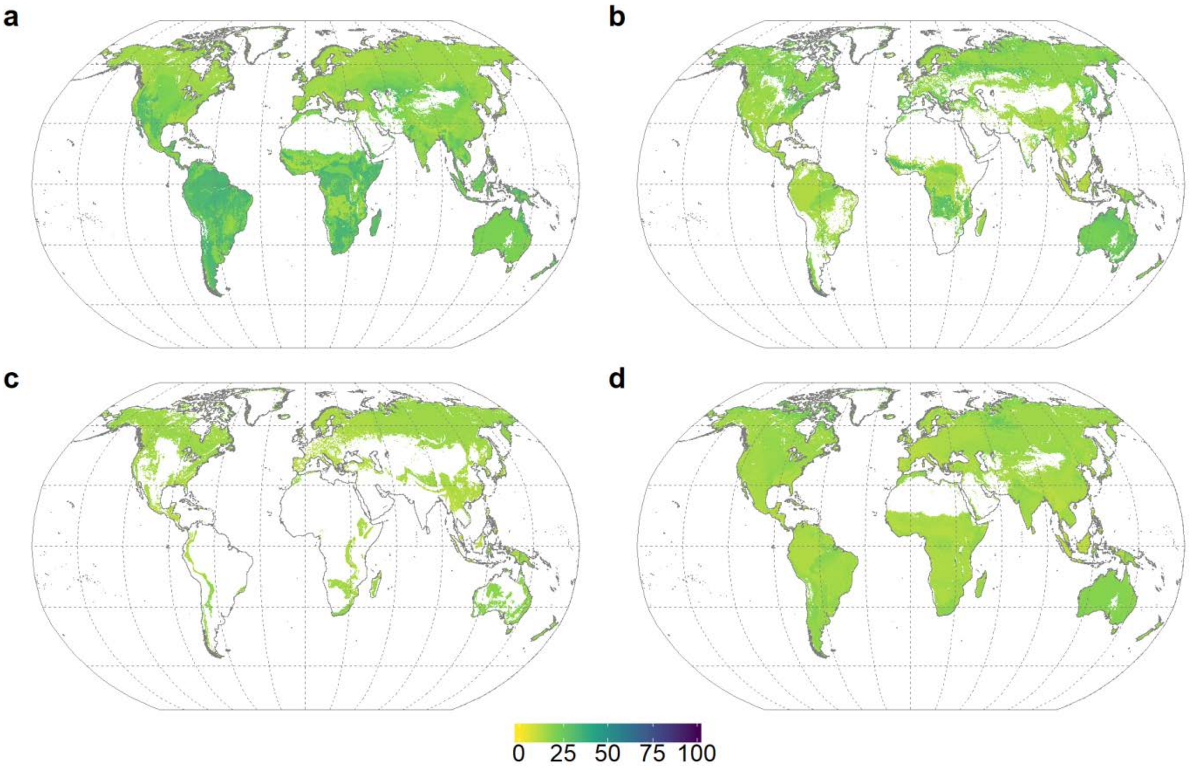


**Supplementary Figure 4. Uncertainty in the percentage of aboveground mycorrhizal plant biomass. a - arbuscular mycorrhizal plants, b - ectomycorrhizal plants, c - ericoid mycorrhizal plants, and d -non-mycorrhizal plants. Colour intensity reflects uncertainty at 90% CI of the biomass fraction of mycorrhizal plants of a given type in a grid cell of 10 arcmin. Source data are provided as a Source Data file.**

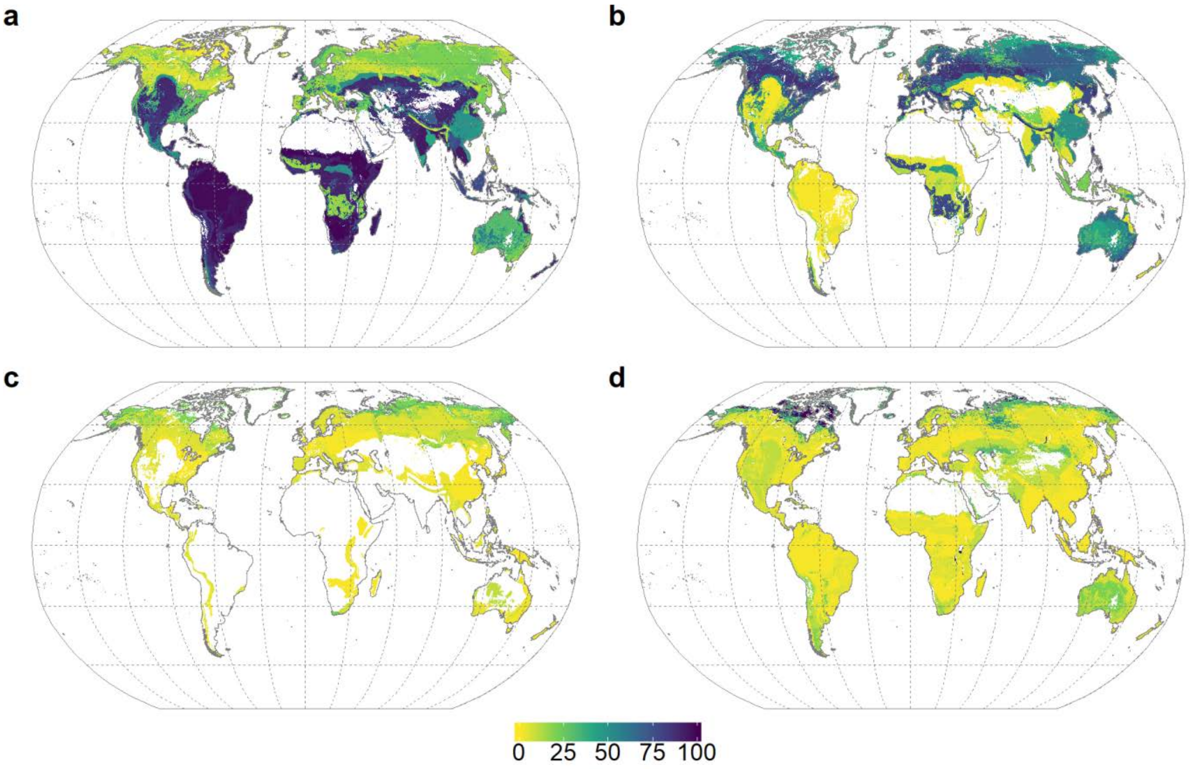


**Supplementary Figure 5. Potential distribution of mycorrhizal vegetation in a cropland-free world. a - arbuscular mycorrhizal plants, b - ectomycorrhizal plants, c - ericoid mycorrhizal plants, and –d - non-mycorrhizal plants. Colour intensity reflects biomass fraction of mycorrhizal plants of a given type in a grid cell of 10 arcmin. Source data are provided as a Source Data file.**

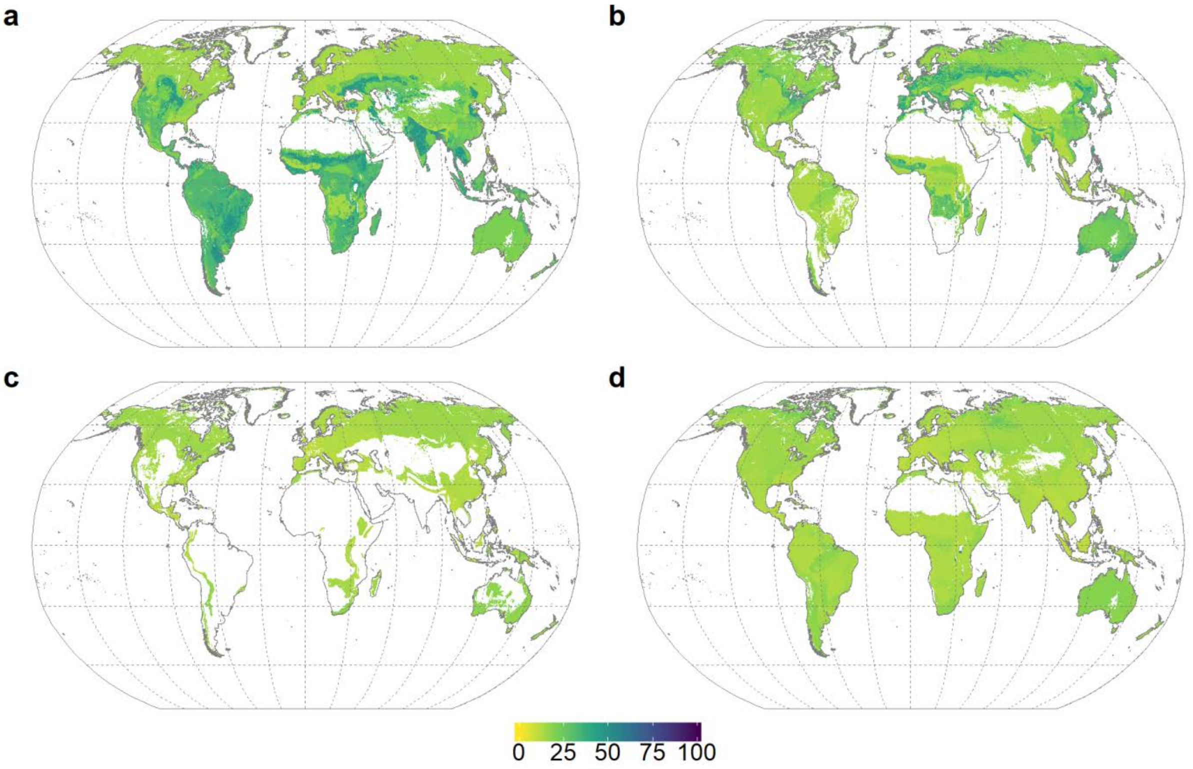


**Supplementary Figure 6. Uncertainty in potential distribution of mycorrhizal vegetation in a cropland-free world. a - arbuscular mycorrhizal plants, b - ectomycorrhizal plants, c - ericoid mycorrhizal plants, and d - non-mycorrhizal plants. Colour intensity reflects uncertainty at 90% confidence interval of the biomass fraction of mycorrhizal plants of a given type in a grid cell of 10 arcmin. Source data are provided as a Source Data file.**

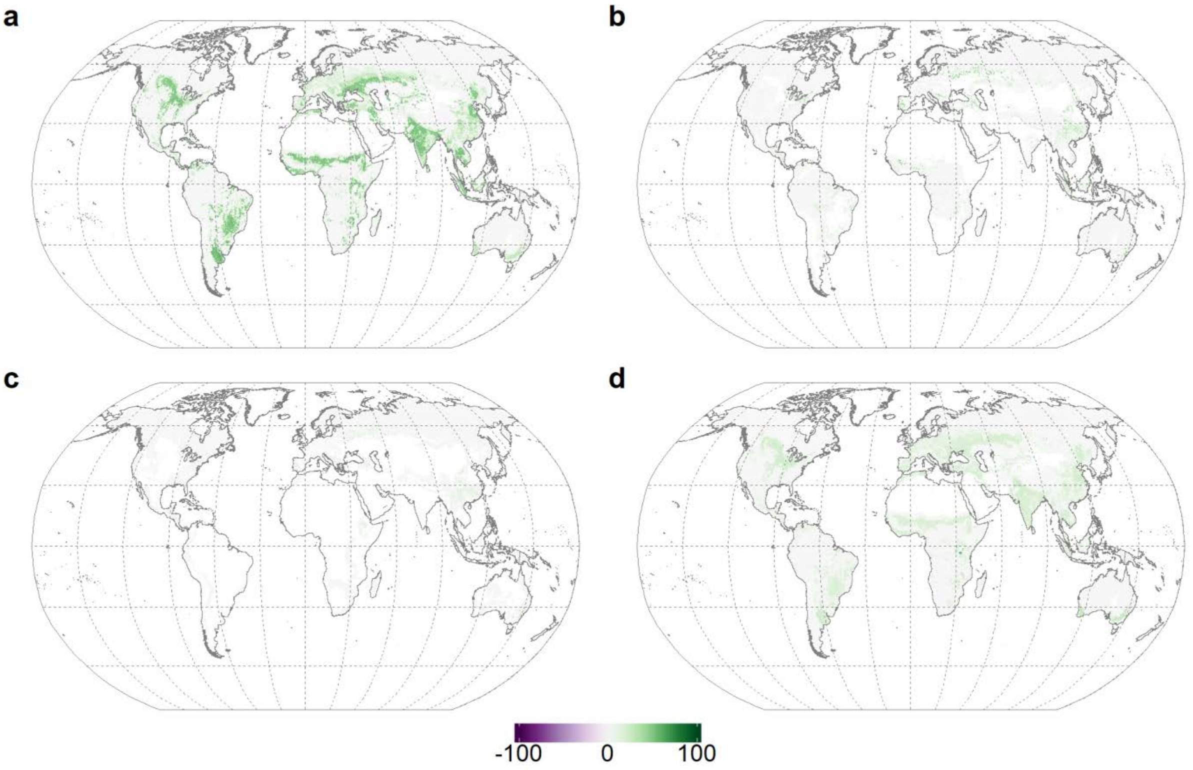


**Supplementary Figure 7. Uncertainty in changes in biomass fractions of mycorrhizal vegetation induced by crop cultivation and pastures. A - arbuscular mycorrhizal plants, b - ectomycorrhizal plants, c - ericoid mycorrhizal plants, d - non-mycorrhizal plants. Purple colors indicate uncertainty in losses of biomass, green colors indicate uncertainty in gains of biomass. Color intensity reflects uncertainty at 90% CI of biomass fraction of mycorrhizal plants of a given type in a grid cell of 10 arcmin. Source data are provided as a Source Data file.**

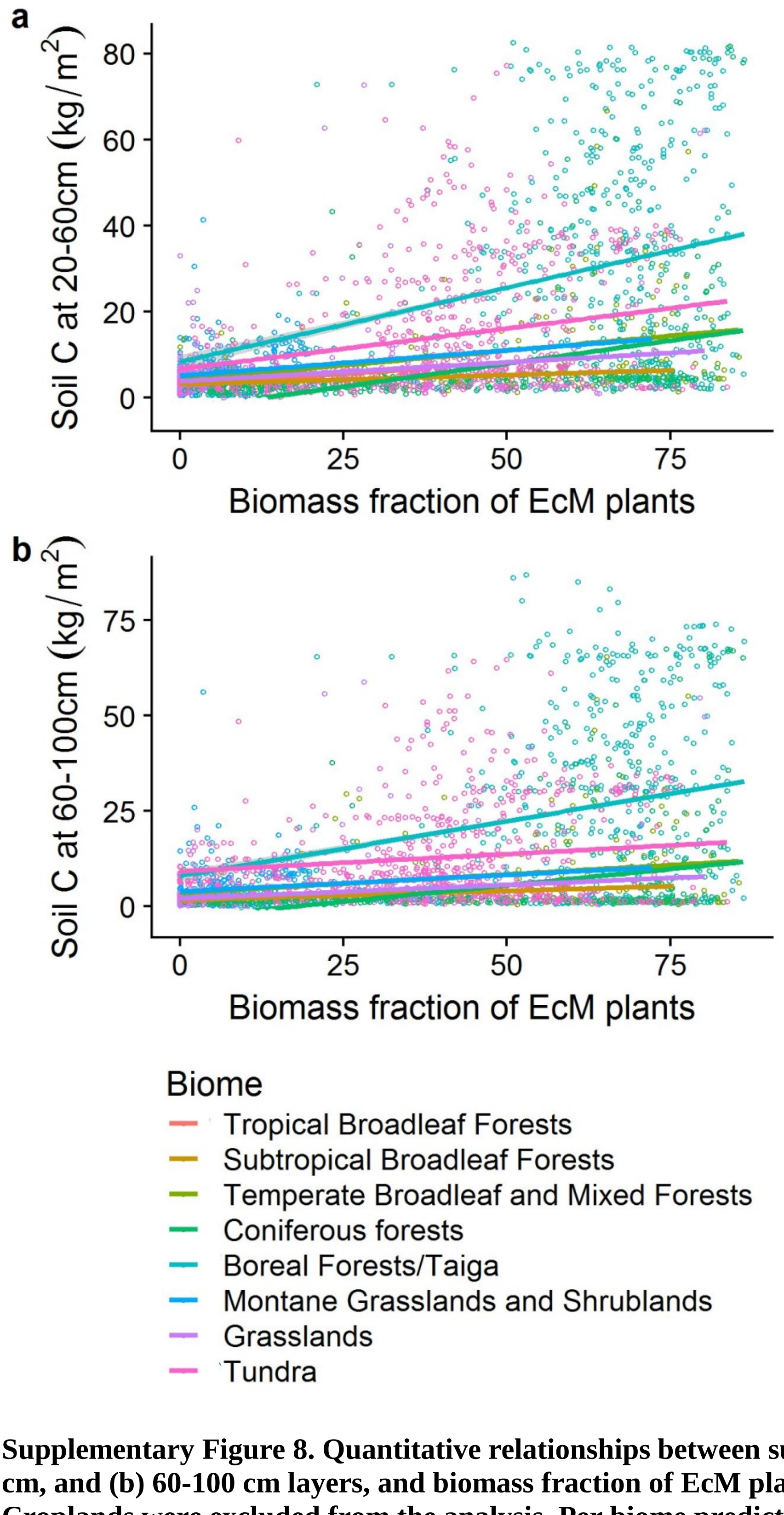


**Supplementary Figure 8. Quantitative relationships between subsoil carbon at (a) 20-60 cm, and (b) 60-100 cm layers, and biomass fraction of EcM plants in an ecosystem. Croplands were excluded from the analysis. Per biome predictions are shown. Source data are provided as a Source Data file.**

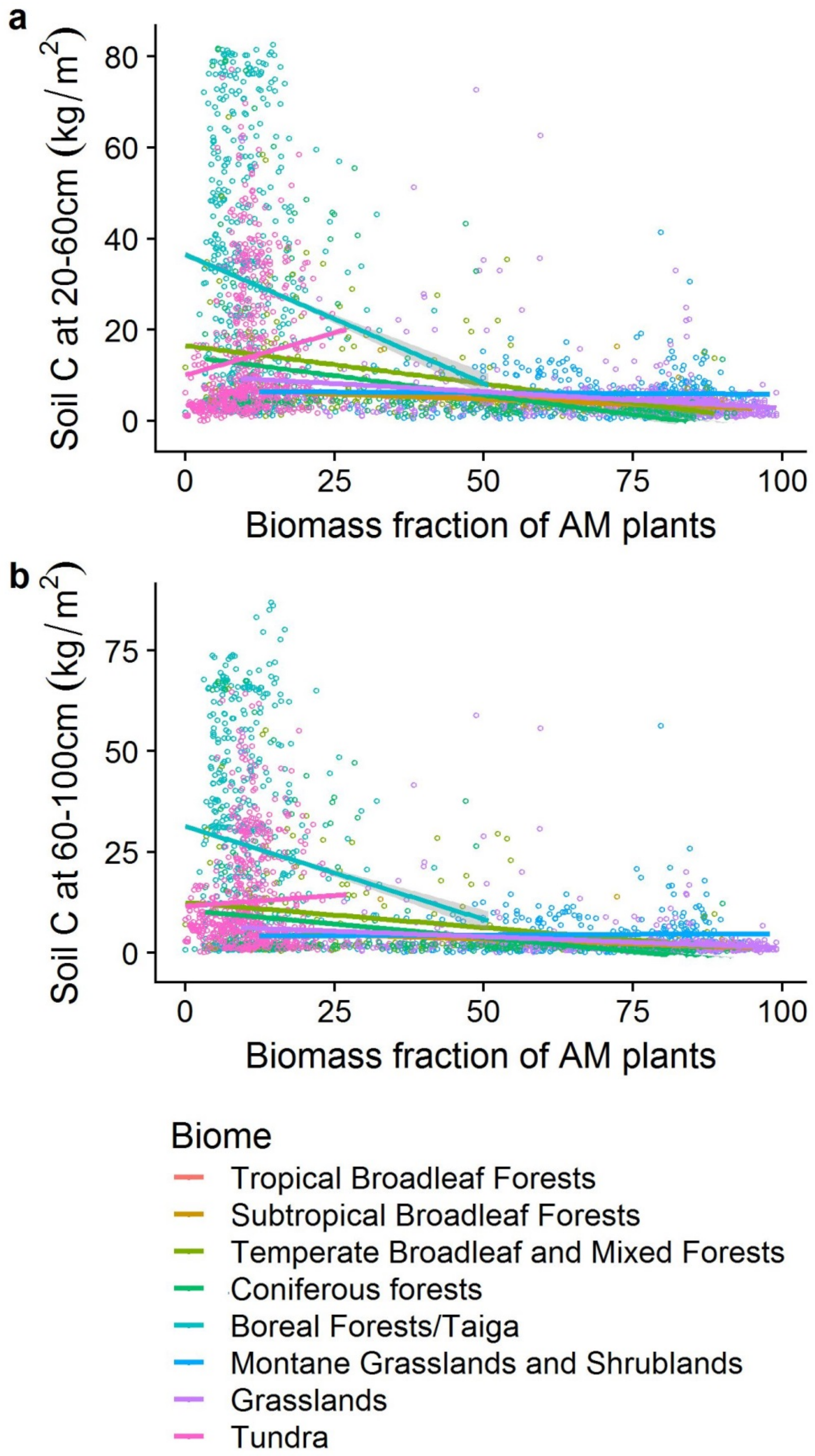


**Supplementary Figure 9. Quantitative relationships between subsoil carbon at (a) 20-60 cm, and (b) 60-100 cm layers, and biomass fraction of AM plants in an ecosystem. Croplands were excluded from the analysis. Per biome predictions are shown. Source data are provided as a Source Data file.**

**Supplementary Table 1. Per biome distribution ± uncertainty at 90% CI of the vegetation carbon stocks among mycorrhizal types. The data presents the sum of carbon stored in vegetation of each mycorrhizal type, across all land cover categories present in each biome, including agricultural, urban and other human-transformed areas.**

| | Absolute values of C stock (GT) | | | | Uncertainty at 90% CI of the absolute values of C stock (GT) | | | |
|---|---|---|---|---|---|---|---|---|
| Biome | AM | EcM | ErM | NM | AM | EcM | ErM | NM |
| Tropical and Subtropical Moist Broadleaf Forests | 142.8 | 14.2 | 1.12 | 8.35 | 8.95 | 3.99 | 0.18 | 1.93 |
| Tropical and Subtropical Dry Broadleaf Forests | 4.10 | 0.51 | 0.02 | 0.86 | 2.61 | 0.86 | 0.03 | 1.21 |
| Temperate Broadleaf and Mixed Forests | 13.5 | 18.2 | 0.68 | 3.48 | 6.85 | 7.85 | 0.35 | 2.97 |
| Boreal Forests/Taiga | 7.30 | 33.5 | 2.97 | 3.47 | 3.28 | 7.30 | 1.58 | 2.76 |
| Montane Grasslands and Shrublands | 2.32 | 0.24 | 0.04 | 0.28 | 2.36 | 0.48 | 0.06 | 0.38 |
| Grasslands | 33.1 | 10.5 | 0.19 | 5.10 | 2.36 | 4.71 | 0.17 | 1.79 |
| Tundra | 0.49 | 1.38 | 0.51 | 0.92 | 0.37 | 1.16 | 0.47 | 1.29 |

**Supplementary Table 2. Summary of statistical models predicting soil carbon stocks. Predictions are made for soil C at 0-20, 20-60, and 60-100 cm depth based on the fractions of EcM or AM plants in vegetation biomass within individual biomes as provided by mycorrhizal vegetation maps, Coeff. – slope as revealed by a generalized linear model (glm), SE – standard error of the slope. Source data are provided as a Source Data file.**

| | | 0-20 cm soil | | | | 20-60 cm soil | | | | 60-100 cm soil | | | |
|---|---|---|---|---|---|---|---|---|---|---|---|---|---|
| Biome | | $R^2$ | P | Coeff. | SE | $R^2$ | P | Coeff. | SE | $R^2$ | P | Coeff | SE |
| Tropical Broadleaf Forests | EcM | 0.49 | <0.001 | 40.2 | 3.9 | 0.77 | <0.001 | 83.3 | 4.3 | 0.94 | <0.001 | 78.0 | 1.9 |
| | AM | 0.45 | <0.001 | --26.5 | 2.8 | 0.68 | <0.001 | -53.7 | 3.5 | 0.95 | <0.001 | -54.1 | 1.2 |
| Subtropical Broadleaf Forests | EcM | 0.14 | <0.001 | 33.0 | 2.2 | 0.08 | <0.001 | 38.4 | 3.6 | 0.12 | <0.001 | 43.4 | 3.1 |
| | AM | 0.18 | <0.001 | -33.8 | 1.9 | 0.09 | <0.001 | -37.2 | 3.2 | 0.14 | <0.001 | --40.9 | 2.8 |
| Temperate Broadleaf and Mixed Forests | EcM | 0.26 | <0.001 | 140.6 | 3.4 | 0.07 | <0.001 | 131.2 | 7.1 | 0.04 | <0.001 | 91.5 | 6.7 |
| | AM | 0.34 | <0.001 | -177.0 | 3.5 | 0.09 | <0.001 | -16.6 | 7.7 | 0.05 | <0.001 | -121.7 | 7.5 |
| Coniferous forests | EcM | 0.28 | <0.001 | 140.4 | 3.0 | 0.09 | <0.001 | 182.2 | 7.9 | 0.07 | <0.001 | 144.0 | 6.9 |
| | AM | 0.26 | <0.001 | -131.7 | 3.0 | 0.06 | <0.001 | -148.4 | 7.7 | 0.05 | <0.001 | -116.0 | 6.8 |
| Boreal Forests/Taiga | EcM | 0.01 | <0.001 | 75.7 | 4.2 | 0.02 | <0.001 | 164.4 | 11.3 | 0.02 | <0.001 | 215.1 | 10.6 |
| | AM | 0.03 | <0.001 | -294.8 | 10.7 | 0.02 | <0.001 | -540.0 | 29.0 | 0.01 | <0.001 | -458.7 | 17.0 |
| Montane Grasslands and Shrublands | EcM | 0.18 | <0.001 | 191.5 | 3.9 | 0.05 | <0.001 | 111.2 | 5.1 | 0.02 | <0.001 | 81.2 | 5.6 |
| | AM | 0.03 | <0.001 | -37.70 | 2.3 | 0.00 | 0.574 | 1.5 | 2.7 | 0.00 | <0.001 | 15.9 | 3.0 |
| Grasslands | EcM | 0.06 | <0.001 | 58.9 | 1.6 | 0.05 | <0.001 | 82.9 | 2.5 | 0.06 | <0.001 | 68.6 | 2.0 |
| | AM | 0.06 | <0.001 | -47.5 | 1.3 | 0.06 | <0.001 | -67.9 | 2.0 | 0.06 | <0.001 | -52.1 | 1.6 |
| Tundra | EcM | 0.12 | <0.001 | 132.9 | 2.9 | 0.05 | <0.001 | 165.6 | 5.9 | 0.01 | <0.001 | 80.1 | 5.3 |
| | AM | 0.13 | <0.001 | 481.1 | 10.3 | 0.02 | <0.001 | 372.0 | 20.8 | 0.00 | <0.001 | 134.1 | 18.5 |

**Supplementary Table 3. Areas occupied by tree plantations of distinct mycorrhizal types. For the entire cover of unspecified tree species the OECD FAO (Organization for Economic Co-operation and Development of Food and Agriculture Organization of the United Nations) report[6] provides an estimate 0.536 mln km$^2$. In this analysis we have split this area between EcM tree plantations and AM tree plantations.**

| | Area mln km$^2$ | Data source |
|---|---|---|
| Global area plantations EcM trees | | |
| *Pinus* spp. (pines) | 0.400 | *6* |
| Other coniferous trees (mostly EcM) | 0.274 | *6* |
| Unspecified tree species | 0.268 | *6* |
| Total | 0.742 | |
| Global area plantations AM/EcM trees | | |
| *Eucalyptus* spp. (eucalypts) | 0.200 | *6* |
| *Acacia spp. (wattles)* | 0.083 | *6* |
| Total | 0.283 | |
| Global area plantations AM trees | | |
| *Elaeis guineensis* (oil palm) | 0.170 | *7* |
| *Hevea brasilensis* (rubber tree) | 0.098 | *6* |
| *Tectona grandis* (teak) | 0.057 | *6* |
| Other broadleaf tree species (mostly AM) | 0.335 | *6* |
| Unspecified tree species | 0.268 | *6* |
| Total | 0.928 | |

**Supplementary Table 4. Summary of the outcomes of an additional regression analysis on the relationship between soil C the fraction of EcM or AM plants in vegetation biomass using Model II regression.**

| Response Variable | Predictors | Slope | P-value slope | $R^2$ |
|---|---|---|---|---|
| C in top soil (0-20 cm) | EM | 0.309 | <0.001 | 0.41 |
| | EM and Biome | Varies per biome, but is always positive | | |
| | AM | -0.275 | <0.001 | 0.39 |
| | EM and Biome | Varies per biome, having ranges from positive to negative | | |
| C at 20-60 cm soil depth | EM | 0.623 | <0.001 | 0.24 |
| | EM and Biome | Varies per biome, but is always positive | | |
| | AM | -0.556 | <0.001 | 0.19 |
| | EM and Biome | Varies per biome, having ranges from positive to negative | | |
| C at 60-100cm soil depth | EM | 0.57 | <0.001 | 0.21 |
| | EM and Biome | Varies per biome, but is always positive | | |
| | AM | -0.509 | <0.001 | 0.19 |
| | EM and Biome | Varies per biome, having ranges from positive to negative | | |

**Supplementary Table 5. Summary of generalized least squares (gls) statistical models predicting soil carbon stocks, and accounting for autocorrelations. Predictions are done for soil C at 0-20, 20-60, and 60-100 cm depth and are based on biome and fraction of EcM or AM plants in vegetation biomass. We used the gls formula "correlation = corExp(form = ~y + x)" Each model has been run 100 times on a selection of 2% of the data points of the entire dataset. The value shown in the table are the mean values of the 100 runs. Degrees of freedom: $DF_{mycorrhizal\ type}$=1, $DF_{Biome}$=7, $DF_{Biome\ x\ mycorrhizal\ type}$ =7, $DF_{residuals}$ = 1608. Source data are provided as a Source Data file.**

| Predicted Variable | Model | Predictor | P value | F value |
|---|---|---|---|---|
| Topsoil C 0-20cm | EcM + Biome + EcM x Biome | EcM | <0.001 | 96.3 |
| | | Biome | 0.011 | 5.3 |
| | | EcM x Biome | ns | 2.7 |
| Subsoil C 20-60cm | EcM + Biome + EcM x Biome | EcM | <0.001 | 61.1 |
| | | Biome | 0.016 | 4.0 |
| | | EcM x Biome | ns | 2.2 |
| Subsoil C 60-100cm | EcM + Biome + EcM x Biome | EcM | <0.001 | 51.1 |
| | | Biome | 0.006 | 4.8 |
| | | EcM x Biome | ns | 1.7 |
| Topsoil C 0-20cm | AM + Biome + AM x Biome | AM | <0.001 | 37.1 |
| | | Biome | 0.001 | 7.2 |
| | | AM x Biome | 0.001 | 5.8 |
| Subsoil C 20-60cm | AM + Biome + AM x Biome | AM | <0.001 | 19.5 |
| | | Biome | 0.001 | 5.7 |
| | | AM x Biome | 0.034 | 3.1 |
| Subsoil C 60-100cm | AM + Biome + AM x Biome | AM | <0.001 | 24.2 |
| | | Biome | 0.001 | 5.9 |
| | | AM x Biome | ns | 2.6 |

**References to the Supplementary Information**


1 Schepaschenko, D. *et al.* A dataset of forest biomass structure for Eurasia. *Scientific data* **4**, 170070 (2017).

2 Lin, G., McCormack, M. L., Ma, C. & Guo, D. Similar below-ground carbon cycling dynamics but contrasting modes of nitrogen cycling between arbuscular mycorrhizal and ectomycorrhizal forests. *New Phytologist* **213**, 1440-1451 (2017).

3 Fisher, J. B. *et al.* Tree–mycorrhizal associations detected remotely from canopy spectral properties. *Global Change Biology* (2016).

4 Brundrett, M. C. in *Biogeography of Mycorrhizal Symbiosis Ecol. Stud.* (ed L. Tedersoo) Ch. 17, 361-394 (2017).

5 Pontius, R. G., Thontteh, O. & Chen, H. Components of information for multiple resolution comparison between maps that share a real variable. *Environmental and Ecological Statistics* **15**, 111-142 (2008).

6 FAO. Global forest resource assessement. (2000).

7. FAO. *Oilseeds and oilseed products. OECD-FAO Agricultural Outlook.* (2013).
