# Supplementary material for: Global mycorrhizal plant distribution linked to terrestrial carbon stocks
Source: Nat Commun. 2019 Nov 7;10:5077. doi: 10.1038/s41467-019-13019-2 (PMC6838125; doi:10.1038/s41467-019-13019-2)
Supplement: Supplementary file 3 — Description of Additional Supplementary Files [file 41467_2019_13019_MOESM3_ESM.pdf]

Description of Additional Supplementary Files

File Name: Supplementary Data 1
Description: List of Bailey ecoregions used to assemble maps of mycorrhizal plants distribution.

File Name: Supplementary Data 2
Description: List of land cover categories within the ESA CCI Land Cover dataset, used to assemble maps of mycorrhizal plants distribution.

File Name: Supplementary Data 3
Description: Literature sources used in assessment of plant community composition across combinations of ecoregions, continents and landcover types.

File Name: Supplementary Data 4
Description: Literature sources used in assessment of mycorrhizal association type of plant species.

File Name: Supplementary Data 5
Description: Mycorrhizal plant cover data used for mapping of current time continental mycorrhizal vegetation.

File Name: Supplementary Data 6
Description: Mycorrhizal plant cover data used for mapping of current time mycorrhizal vegetation on islands

File Name: Supplementary Data 7
Description: Mycorrhizal plant cover data used for mapping of continental mycorrhizal vegetation in a cropland-free world.

File Name: Supplementary Data 8
Description: Mycorrhizal plant cover data used for mapping of island mycorrhizal vegetation in a cropland-free world
