# Supplementary material for: Global mycorrhizal plant distribution linked to terrestrial carbon stocks
Source: Nat Commun. 2019 Nov 7;10:5077. doi: 10.1038/s41467-019-13019-2 (PMC6838125; doi:10.1038/s41467-019-13019-2)
Supplement: Supplementary file 12 — Reporting Summary [file 41467_2019_13019_MOESM12_ESM.pdf]

natureresearch

Corresponding author(s): Nadejda A. Soudzilovskaia ; manuscript NCOMMS-18-33964

Last updated by author(s): Sep 20, 2019

Reporting Summary

Nature Research wishes to improve the reproducibility of the work that we publish. This form provides structure for consistency and transparency in reporting. For further information on Nature Research policies, see Authors & Referees and the Editorial Policy Checklist.

Statistics

For all statistical analyses, confirm that the following items are present in the figure legend, table legend, main text, or Methods section.

n/a	Confirmed	
☐	☒	The exact sample size (*n*) for each experimental group/condition, given as a discrete number and unit of measurement
☒	☐	A statement on whether measurements were taken from distinct samples or whether the same sample was measured repeatedly
☐	☒	The statistical test(s) used AND whether they are one- or two-sided<br>*Only common tests should be described solely by name; describe more complex techniques in the Methods section.*
☐	☒	A description of all covariates tested
☒	☐	A description of any assumptions or corrections, such as tests of normality and adjustment for multiple comparisons
☐	☒	A full description of the statistical parameters including central tendency (e.g. means) or other basic estimates (e.g. regression coefficient) AND variation (e.g. standard deviation) or associated estimates of uncertainty (e.g. confidence intervals)
☐	☒	For null hypothesis testing, the test statistic (e.g. *F*, *t*, *r*) with confidence intervals, effect sizes, degrees of freedom and *P* value noted<br>*Give P values as exact values whenever suitable.*
☒	☐	For Bayesian analysis, information on the choice of priors and Markov chain Monte Carlo settings
☒	☐	For hierarchical and complex designs, identification of the appropriate level for tests and full reporting of outcomes
☐	☒	Estimates of effect sizes (e.g. Cohen's *d*, Pearson's *r*), indicating how they were calculated

Our web collection on statistics for biologists contains articles on many of the points above.

Software and code

Policy information about availability of computer code

Data collection	Internet Explorer browther, google scholar and ISI Web of Science search engines were used to collect the data on mycorrhizal vegegtaion underlying the maps presented in the manuscript
Data analysis	All data processing and analsyis was conducted using R statistical software (R R Foundation for Statistical Computing, Vienna, Austria). All R scripts used to create maps of mycorrhizal vegegtation and the dataset used to analyze the relationships between data of mycorrhizal vegetation maps and above and belowground soil C stocks are available at https://github.com/nasoudzilovskaia/Soudzilovskaia_NatureComm_MycoMaps.

For manuscripts utilizing custom algorithms or software that are central to the research but not yet described in published literature, software must be made available to editors/reviewers. We strongly encourage code deposition in a community repository (e.g. GitHub). See the Nature Research guidelines for submitting code & software for further information.

Data

Policy information about availability of data

All manuscripts must include a data availability statement. This statement should provide the following information, where applicable:

- Accession codes, unique identifiers, or web links for publicly available datasets
- A list of figures that have associated raw data
- A description of any restrictions on data availability

The maps of mycorrhizal vegetation are freely available on request to the corresponding author, and at https://github.com/nasoudzilovskaia/Soudzilovskaia_NatureComm_MycoMaps. The original datasets used to generate the maps of mycorrhizal vegetation are available as Supplementary Data Tables 5-8. All the links to the publically available datasets, such as map of Bailey ecoregions, map of Earth continents and map of landcover types are provided in the reference list of the manuscript.

Field-specific reporting

Please select the one below that is the best fit for your research. If you are not sure, read the appropriate sections before making your selection.

☐ Life sciences ☐ Behavioural & social sciences ☒ Ecological, evolutionary & environmental sciences

For a reference copy of the document with all sections, see nature.com/documents/nr-reporting-summary-flat.pdf

Ecological, evolutionary & environmental sciences study design

All studies must disclose on these points even when the disclosure is negative.

Study description	Based on a comprehensive quantitative evaluation of plant-mycorrhizal associations and the distribution of vascular plant species across biomes and continents available formpublished datasets, we assembled the first high-resolution digital maps of the global distribution of biomass fractions of AM, EcM, ErM, and NM plants. Building on these maps, we assessed (i) the amount of aboveground biomass carbon currently stored in each type of mycorrhizal vegetation, (ii) the impact of conversion of natural ecosystems to croplands on the distribution of mycorrhizal types globally, and (iii)the difference in relationships between relative abundance of AM and EcM plants in an ecosystem and ecosystem soil carbon content in topsoil (0-20cm), medium (20-60) and deep (60-100 cm) subsoil layers. . The questions I and II are answered by calculations, i.e. no statitical analysis is involved. The question III is answered with linear model regression analyses, using soil C data from the ISRIC-WISE soil dataset as responce variable and the data of biome type and biomas fractions of AM and EM plants per unit area, as predictors. The data of biomas fractions of AM and EM plants per unit area is taken from the maps of mycorrhizal vegegtation generated within this study. The data of biome types and soil C are taken form the publically available datasets. The Internet links to the latter are provided in the manusciprit text.
Research sample	The research sample constitutes the data of mycorrhizal vegetation maps generated within this study (see study description for details).
Sampling strategy	N/A
Data collection	The data underlying the mycorrhizal vegetation maps was collected by Nadejda A. Soudzilovskaia and Leho Tedersoo, with assistance of PhD and MSc students of their research intitutions. Involved students are acknowlenged in the manuscript.
Timing and spatial scale	The data was collected from the published datasets available via the Internet . Th time frame: 1930 - 2019; Spatial scale: global
Data exclusions	All available data was used to generate the maps of mycorrhizal vegetation. The impact of conversion of natural ecosystems to croplands on the distribution of mycorrhizal types globally was assessed on the entire maps. The difference in relationships between relative abundance of AM and EcM plants in an ecosystem and soil carbon content was analysed based on the map points representinig natural and biome-typical vegetation. The assessemnt of adta suitabillity was done based on teh ESA gloval landcover dataset (2015), and biomes map of FAO
Reproducibility	N/A
Randomization	N/A
Blinding	N/A

Did the study involve field work? ☐ Yes ☒ No

Reporting for specific materials, systems and methods

We require information from authors about some types of materials, experimental systems and methods used in many studies. Here, indicate whether each material, system or method listed is relevant to your study. If you are not sure if a list item applies to your research, read the appropriate section before selecting a response.

Materials & experimental systems

n/a	Involved in the study	
☒	☐	Antibodies
☒	☐	Eukaryotic cell lines
☒	☐	Palaeontology
☒	☐	Animals and other organisms
☒	☐	Human research participants
☒	☐	Clinical data

Methods

n/a	Involved in the study	
☒	☐	ChIP-seq
☒	☐	Flow cytometry
☒	☐	MRI-based neuroimaging
